# Association Between Sarcopenic Complications and Readmission in a Kaifukuki Rehabilitation Ward: A Retrospective Cohort Study

**DOI:** 10.7759/cureus.50067

**Published:** 2023-12-06

**Authors:** Tomoki Hayashi, Yuki Kataoka

**Affiliations:** 1 Department of Rehabilitation, Kyoto Min-iren Asukai Hospital, Kyoto, JPN; 2 Section of Clinical Epidemiology, Department of Community Medicine, Kyoto University Graduate School of Medicine, Kyoto, JPN; 3 Department of Healthcare Epidemiology, Kyoto University Graduate School of Medicine, School of Public Health, Kyoto, JPN; 4 Department of Systematic Reviewers, Scientific Research WorkS Peer Support Group, Kyoto, JPN; 5 Department of Internal Medicine, Kyoto Min-iren Asukai Hospital, Kyoto, JPN; 6 Department of Systematic Reviewers, Kyoto Min-iren Asukai Hospital, Kyoto, JPN

**Keywords:** elderly inpatient, retrospective cohort study, readmission, sarcopenia, kaifukuki rehabilitation ward

## Abstract

Objectives

This study investigated the association between sarcopenia and readmission to the Kaifukuki Rehabilitation Ward.

Methods

We conducted a retrospective observational study in a Kaifukuki Rehabilitation Ward in Japan. Muscle mass was evaluated using a body composition analyzer (InBody, Tokyo, Japan). Grip strength was measured using a grip dynamometer, and walking speed was measured using a 10-meter walk test. Sarcopenia was characterized based on the diagnostic algorithm recommended by the Asian Working Group for Sarcopenia. The presence or absence of readmission was calculated from the medical charts. This study used Rstudio for statistical analysis (Posit, Boston, USA). To examine the effect of sarcopenia on readmissions, we used the Kaplan-Meier method to estimate readmissions. Differences between curves were assessed using the log-rank test.

Results

A total of 131 patients were selected during the target period (March 1, 2020, to August 31, 2021). Of these, 12 (9%) were readmitted during the study period. The median patient age was 83 years. The study population consisted of 53 males (40%) and 78 females (60%). Sixty (50%) patients in the no-readmission group and seven (58%) patients in the readmission group had sarcopenia. For readmission, the presence of sarcopenia yielded an unadjusted hazard ratio of 1.37 (95% confidence interval: 0.41 to 4.56) and an adjusted hazard ratio of 1.74 (95% confidence interval: 0.52 to 5.83).

Conclusions

Sarcopenia may be a prognostic factor for readmission in Kaifukuki Rehabilitation Wards. Therefore, further evaluation is necessary.

## Introduction

Sarcopenia, characterized as the decline in muscle mass, strength, and function associated with aging is now regarded as a novel geriatric condition [1]. Evidence suggests that sarcopenia can develop rapidly after acute illness and that the rate of change in skeletal muscle mass itself predicts poor prognosis [2-3]. Studies have reported that the frequency of sarcopenia ranges from 1-29% among those living in communities, 14-33% among those in long-term care settings, and 10% in acutely admitted to hospital populations [4].

Prior research has shown that sarcopenia is strongly linked to recurrent falls, fractures, diminished functionality, disability, admissions to hospitals and nursing homes, reduced quality of life, and mortality. Reports have shown that sarcopenia is a factor that affects mortality and readmission rates three years after discharge [5]. However, previous studies have focused on community-dwelling elderly people, facility residents, and patients in acute wards, and there have been no studies on patients in Kaifukuki rehabilitation wards (KRWs).

The KRW primarily provides intensive rehabilitation to facilitate the recovery of bedridden elderly people and support their return home. It is a specialized ward with clear regulations on various aspects such as length of stay and target diseases, and is unique to Japan [6-7]. The KRW provides intensive rehabilitation based on interprofessional collaboration to promote the reintegration of patients into a state of physical and mental recovery [8]. However, many of the patients who were discharged from the KRW showed demerit points in Functional Independence Measure (FIM) motor items compared to when they were admitted, and physical function deteriorated over time in patients with stroke [9-10]. Decreased physical function leads to inactivity, which is expected to cause further deterioration. There is a risk that this will lead to a decline in activities of daily living/quality of life (ADL/QOL), an increase in falls, and readmission. Therefore, this study aimed to describe the presence of sarcopenia in KRW patients, investigate the association between sarcopenia and readmission, and explore other predictors of readmission.

## Materials and methods

This was a retrospective cohort study conducted on patients in the 51 beds at the KRW at Kyoto Min-iren Asukai Hospital. The Ethics Committee of the Kyoto Min-iren Central Hospital approved the study protocol to gain opt-out consent from the patients (ID 137). We report this study using the STrengthening the Reporting of OBservational studies in Epidemiology (STROBE) guidelines (Table 1).

**Table 1:** STrengthening the Reporting of OBservational studies in Epidemiology (STROBE) checklist

	Item No	Recommendation	PageNo
Title and abstract	1	(a) Indicate the study’s design with a commonly used term in the title or the abstract	2
		(b) Provide in the abstract an informative and balanced summary of what was done and what was found	2
Introduction			
Background/rationale	2	Explain the scientific background and rationale for the investigation being reported	3
Objectives	3	State specific objectives, including any prespecified hypotheses	3
Methods			
Study design	4	Present key elements of address the study design at the outset of the paper.	4
Setting	5	Describe the setting, locations, and pertinent timeframes, encompassing recruitment phases, exposure periods, follow-up intervals, and data collection epochs.	4-5
Participants	6	(a) Cohort study—Give the eligibility criteria, and the sources and approaches for Participant Selection. Describe methods of follow-up Case-control study—Give the eligibility criteria, and the sources and methods of case ascertainment and control selection. Provide the reasoning behind selecting specific cases and controls in a cross-sectional study outline the criteria for eligibility., and the sources and methods of selection of participants	4
		(b) Cohort study—For matched studies, give matching criteria and number of exposed and unexposed Case-control study—For matched studies, give matching criteria and the number of controls per case	4
Variables	7	Clearly define all outcomes, exposures, predictors, potential confounders, and effect modifiers. Give diagnostic criteria, if applicable	4
Data sources/measurement	8*	For each variable of interest, give sources of data and details of methods of assessment (measurement). Describe comparability of assessment methods if there is more than one group	4-5
Bias	9	Describe any efforts to address potential sources of bias	NA
Study size	10	Explain how the study size was arrived at	4[YK1]
Quantitativevariables	11	Explain how quantitative the analyses included the consideration of variables. If applicable, please elucidate the selected groupings and provide rationale for their choice.	4
Statistical methods	12	(a) Describe all statistical methods, incorporating methods employed to account for confounding factors.	5
		(b) Describe any methods commonly employed for the analysis of subgroups and interactions.	5-6
		(c) Explain how missing data were addressed	5[YK2]
		(d) Cohort study—If applicable, explain how loss to follow-up was addressed Case-control study—If applicable, explain how matching of cases and controls was addressed Cross-sectional study—If applicable, describe analytical methods taking account of sampling strategy	4[YK3]
		(e) Describe any sensitivity analyses	NA

Results			
Participants	13*	(a) Report numbers of individuals at every phase of the research, such as assessing potentially eligible individuals, scrutinizing eligibility, confirming eligibility, incorporating participants into the study, conducting follow-up, and performing analysis.	6
		(b) Give reasons for non-participation at each stage	6
		(c) Consider use of a flow diagram	13
Descriptive data	14*	(a) Give characteristics of study subjects, encompassing demographic, clinical, and social factors, alongside details regarding exposures and potential confounding variables.	6
		(b) Indicate number of participants with missing data for each variable of interest	NA
		(c) Cohort study—Summarise follow-up time (eg, average and total amount)	6
Outcome data	15*	Cohort study—Report numbers of outcome events or summary measures over time	6[YK4]
		Case-control study—Report numbers in each exposure category, or summary measures of exposure	NA
		Cross-sectional study—Report numbers of outcome events or summary measures	NA
Main results	16	(a) Give unadjusted estimates and, If relevant, provide adjusted estimates accounting for confounding factors along with their precision, such as the 95% confidence interval. Clearly specify the confounding variables that were taken into account and elucidate the rationale for their inclusion.included	6-7
		(b) Report category boundaries when continuous variables were categorized	NA
		(c) If relevant, consider translating estimates of relative risk into absolute risk for a meaningful time period	NA
Other analyses	17	Report other analyses done—eg analyses of subgroups and interactions, and sensitivity analyses	6
Discussion			
Key results	18	Summarise key results with reference to study objectives	7
Limitations	19	Discuss limitations of the study, consider potential sources of bias and inaccuracy. We investigate both the direction and magnitude of potential bias.	8-9
Interpretation	20	Give a cautious overall interpretation of results considering objectives, limitations, multiplicity of analyses, results from similar studies, and other relevant evidence	7-8
Generalisability	21	Discuss the generalisability (external validity) of the study results	8
Other information			
Funding	22	Give the source of funding and the role of the funders for the present study and, if applicable, for the original study on which the present article is based	9

Sample size

We set the sample size based on the data extraction capability of InBody. There were no software or formal specifications for determining sample size.

Study population

The participants were patients who were admitted to the Kaifukuki rehabilitation ward of Kyoto Min-iren Asukai Hospital between December 1, 2016, and August 31, 2022, and were discharged. Information was collected from medical charts, and participants were selected for whom information necessary for diagnosing sarcopenia, such as grip strength, walking speed, and skeletal muscle mass, could be extracted, and for whom follow-up was possible after discharge.

Data collection

We collected data on height, age, sex, grip strength, walking speed, skeletal muscle mass, nutritional status (albumin), length of hospital stay, main disease (disease classification), and whether or not the patient was readmitted from the chart.

Assessment of sarcopenia

The criteria listed in the Asian Working Group for Sarcopenia 2019 (AWGS2019) were used to define sarcopenia in this study [11]. Grip strength measurement: men <28 kg, women <18 kg; walking speed <1.0 m/s or less; and skeletal muscle mass index (SMI) measured by the bioelectrical impedance method (BIA) <7.0 kg/m2 for men and <5.7 kg/m2 for women. Those with less than these measurements were diagnosed with sarcopenia. Walking speed and grip strength data were extracted from the charts. SMI was calculated and determined from the records of a body composition analyzer (Inbody430; InBody Japan Inc, Etoku/Tokyo, Japan), which used a tetrapolar eight-point tactile electrode system [12].

The impedance of the system was measured individually on participants' right arm, left arm, trunk, right leg, and left leg at six different frequencies (1, 5, 50, 250, 500, and 1,000  kHz) for every body segment. The participants adhered to the manufacturer's instructions by using a specialized electrolyte tissue to wipe the undersides of their feet prior to stepping onto the electrodes integrated into the scale platform of their analyzers specific to each. The individuals were directed to stand in an upright position and hold onto the analyzer's handles, establishing contact with eight electrodes in the process (two for each foot and hand). From the obtained data, SMI was calculated using the following equation: SMI = total muscle mass of limbs/height × height.

Readmission information

A survey of readmission was conducted based on the medical record information. Starting from the day of discharge, it was confirmed whether the patient had been readmitted to a hospital, including Kyoto Min-iren Asukai Hospital, and the number of days was measured. The end date of the observation period was December 31, 2022.

Statistical analyses

Categorical data are expressed as both absolute values ​​and overall percentages, and continuous data are presented as mean ± standard deviation (SD) if normally distributed. Results expressed as median ± interquartile range were not applicable. There were no missing data in this study. To examine the effect of sarcopenia on readmission, we used the Kaplan-Meier method to estimate readmission. Disparities in the curves were evident-evaluated employing the log-rank test. The adjusted hazard ratios (HRs) and 95% confidence intervals (CIs) for readmission with and without sarcopenia were estimated using Cox proportional hazards models. The following items were used as covariates: age, sex, serum albumin level, length of hospital stay, and diseases that caused hospitalization. We classified these diseases as cerebrovascular diseases, orthopedic diseases, and others. We used RStudio (Posit, Boston, USA) [13].

## Results

Patient characteristics

**Figure 1:**
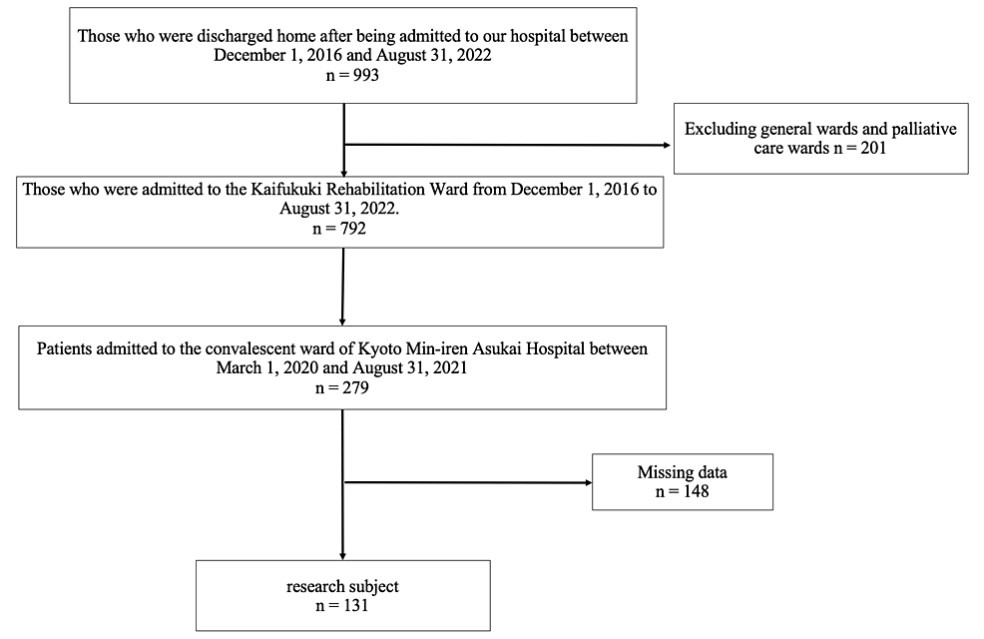
Flowchart for selecting patients for the study

Of the 993 patients who were admitted to our hospital and discharged between December 1, 2016, and August 31, 2022, 201 who were admitted to the general or palliative care ward were excluded. Of the remaining 792 patients, the data of 279 who were hospitalized and discharged during the target period (March 1, 2020, to August 31, 2021) were extracted, and 148 were excluded due to missing data. A total of 131 patients were included in this study (Figure 1). The median observation period was 1.54 years. The clinical characteristics of the study population are summarized in Table 2. The non-readmission group had 45 males (38%) and 74 females (62%), with a median age of 81 years. The re-hospitalized group had eight males (67%) and four females (33%) with a median age of 82 years. Sixty (50%) patients had sarcopenia without readmission, and seven (58%) had sarcopenia in the readmission group. The median length of hospital stay was 74 days in the no-readmission group and 78.5 days in the readmission group (Table 2).

**Table 2:** Characteristics of the study population according to the presence or absence of readmission N: number; *n (%), Median (interquartile range [IQR]), Range

Characteristics	Presence, N = 119^*^	Absence, N = 12^*^
Sex		
Woman	74 (62%)	4 (33%)
Man	45 (38%)	8 (67%)
Disease classification		
Other	2 (1.7%)	2 (1.7%)
Orthopedic disease	66 (55%)	4 (33%)
Cerebrovascular disease	51 (43%)	6 (50%)
Age	82 (74, 87)	84 (78, 87)
Length of stay	70 (48, 86)	79 (53, 97)
Sarcopenia	60 (50%)	7 (58%)

Kaplan-Meier method

**Figure 2:**
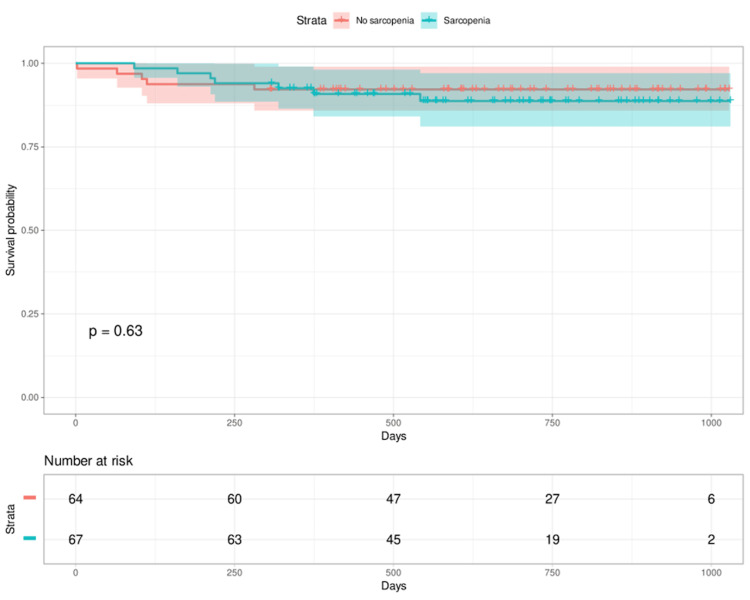
Kaplan–Meier curves for readmission according to the quartiles of the sarcopenia index

Figure 2 shows the Kaplan-Meier curves stratified by the presence or absence of sarcopenia. The median time from discharge to readmission estimated from the Kaplan-Meier curve was not obtained for either the non-sarcopenia group or the sarcopenia group. The survival rate (percentage without readmission) at 750 days was 0.87 in the sarcopenia and 0.93 in the group without sarcopenia. The p-value was 0.63 from the log-rank test.

Cox proportional hazards model

The hazard ratio of readmission risk for sarcopenia (HR 1.74 [95%CI 0.52 to 5.83]) and male sex (HR 3.00 [95%CI 0.18 to 11.1]) were associated with increased readmission risk. Disease classification (orthopedic disease) based on cerebrovascular disease is HR 0.55 (95%CI 0.15 to 2.02), and the disease classification (cerebrovascular disease) based on orthopedic disease was HR 1.79 (95%CI 0.49 to 6.52). The HR for length of stay was 1.01 (95%CI 0.99 to 1.02) and that of age was 1.04 (95%CI 0.97 to 1.11). The readmission risk HR for albumin was 0.28 (95%CI 0.06 to 1.30) (Table 3).

**Table 3:** Cox proportional hazards model

		95% Confidence interval	
	HR	Lower limit	Upper limit
Orthopedic disease (vs. cerebrovascular disease)	0.55	0.15	2.02
Cerebrovascular disease (vs. orthopedic disease)	1.79	0.49	6.52
Length of stay	1.01	0.99	1.02
Sarcopenia	1.74	0.52	5.83
Age	1.04	0.97	1.11
Gender male (vs. female)	3.00	0.81	11.1
Albumin	0.28	0.06	1.30

## Discussion

Summary of the results

We conducted a retrospective cohort study on the KRW. A total of 131 participants were enrolled in the study. Although not statistically significant, sarcopenia was associated with an increased risk of readmission (HR, 1.74 [95% CI 0.52 to 5.83]).

Comparison with previous studies

Sarcopenia may be a predictor of readmission after discharge from the KRW. In a previous acute ward inpatient study, sarcopenia was an independent predictor of readmission 3 years after discharge (adjusted HR: 1.81; 95% CI:1.17-2.80) [5]. A previous study included patients with post-abdominal trauma identified sarcopenia (OR: 2.09, 95% CI: 1.34-3.27) as a significant predictor of 90-day readmission [14]. In this study, the point estimate did not markedly differ from that in previous studies.

Clinical implication

In this study, half of the patients admitted to the KRW had sarcopenia. Given the high prevalence of sarcopenia and its association with worse outcomes among patients in the KRW, early detection and intervention are vital. The presence of sarcopenia should be considered in clinical judgments and intervention measures.

Research implication

Owing to the wide confidence intervals of our results, a larger sample size is needed. In addition, by conducting collaborative research with other medical institutions, it will be possible to include patients of a wide range of ages and diseases in the target population. Moreover, it was necessary to examine the criteria for participant inclusion. In previous studies, the conditions for diagnosing sarcopenia were that walking speed was measurable and that the patient had cognitive functions that enabled communication [5].

Generalisability

It is suggested that the results of this study can be applied to patients in other convalescent rehabilitation units. According to a past national survey by the Association of Convalescent Rehabilitation Wards, as of 2014, 48.0% of hospitalized patients had cerebrovascular diseases and 43.3% had orthopedic diseases [15]. The results of this survey are similar to the disease classification breakdown of the subjects extracted this time.

Limitations

This study has two limitations. First, the timing of sarcopenia diagnosis was not standardized. As this was a retrospective cohort study, the diagnosis of sarcopenia during the hospitalization period was made for each patient based on medical record information, and it was difficult to unify the timing. In the future, we would like to examine the presence or absence of sarcopenia at the time of admission, divide patients into groups, and conduct a prospective study. Second, unmeasured confounders (such as geriatric syndromes, activities of daily living, and interventions) may have biased our results.

## Conclusions

A retrospective cohort study in KRW showed that sarcopenia may be a prognostic factor for readmission. These results suggest the need to monitor patients for the presence of sarcopenia from the time of admission. Further validation using a larger sample size is required to confirm our findings. Furthermore, we believe it is necessary to verify whether preventing and improving sarcopenia from the time of admission has an impact on reducing the risk of readmission.
